# Bidirectional C and N transfer and a potential role for sulfur in an epiphytic diazotrophic mutualism

**DOI:** 10.1038/s41396-020-00738-4

**Published:** 2020-08-19

**Authors:** Rhona K. Stuart, Eric R. A. Pederson, Philip D. Weyman, Peter K. Weber, Ulla Rassmussen, Christopher L. Dupont

**Affiliations:** 1 Physical and Life Sciences Directorate, Lawrence Livermore National Laboratory, Livermore, CA 94550 USA; 2 Department of Ecology, Environment and Plant Sciences, Stockholm University, 106 91 Stockholm, Sweden; 3 J. Craig Venter Institute, La Jolla, CA 92037 USA; 4 Present Address: Zymergen Inc., Emeryville, CA USA

**Keywords:** Microbial ecology, Plant ecology, Biogeochemistry, Stable isotope analysis

## Abstract

In nitrogen-limited boreal forests, associations between feathermoss and diazotrophic cyanobacteria control nitrogen inputs and thus carbon cycling, but little is known about the molecular regulators required for initiation and maintenance of these associations. Specifically, a benefit to the cyanobacteria is not known, challenging whether the association is a nutritional mutualism. Targeted mutagenesis of the cyanobacterial alkane sulfonate monooxygenase results in an inability to colonize feathermosses by the cyanobacterium *Nostoc punctiforme*, suggesting a role for organic sulfur in communication or nutrition. Isotope probing paired with high-resolution imaging mass spectrometry (NanoSIMS) demonstrated bidirectional elemental transfer between partners, with carbon and sulfur both being transferred to the cyanobacteria, and nitrogen transferred to the moss. These results support the hypothesis that moss and cyanobacteria enter a mutualistic exosymbiosis with substantial bidirectional material exchange of carbon and nitrogen and potential signaling through sulfur compounds.

## Introduction

Boreal forest ecosystems cover almost 20% of the Earth’s land mass, store ~30% of terrestrial carbon (C) and nitrogen (N), and play a key role in global C and N biogeochemical cycles [1, 2]. Up to 50% of the N input into boreal forests derives from N-fixation by cyanobacteria living epiphytically on feathermosses such as *Pleurozium schreberi* and *Hylocomium splendens* [3, 4]. There is growing evidence that feathermoss and associated epiphytic cyanobacterial communities are fundamental drivers of boreal forest C and N cycling [4–7]. However, cyanobacterial N-fixation is dynamic, with variability attributed to extrinsic factors such as temperature, light, precipitation, fertilizers, and phylogeny of cyanobacterial communities associated with feathermosses [8–13]. Defining the controls on these epiphytic associations, such as molecular exchanges that regulate their establishment and maintenance, is critical to our ability to predict C and N cycling under dynamic conditions.

For endophytic cyanobacteria-plant symbioses, which are better characterized than epiphytic symbioses, it has been fairly well established that fixed-N products are the main cyanobacterial commodity, and reduced C appears to be best-supported host commodity. These mutualistic symbioses, including angiosperm, hornwort, liverwort and cycad hosts, are mostly facultative [14]. The symbioses form when the host is N-limited, and the symbiotic cyanobacteria release 80–90% of their fixed-N products to the host [15]. It has been shown that reduced C from the plant host fuels at least two-thirds of N-fixation activity between the hornwort *Anthoceros* sp. and *Nostoc* cyanobacterium [15, 16]. In cycad and fern associations, cyanobacterial RubisCO protein abundance and photoautotrophic C fixation activity both decrease when in symbiosis relative to free-living growth [15], again pointing to the role of reduced C, possibly as sugar [17], as the host provided commodity.

For epiphytic associations, there may be similarities, but the picture is less clear. There is evidence that N-fixation is important: fixed-N products from the cyanobacteria are assimilated into the moss biomass, only N-limited moss are colonized [18], and these cyanobacteria induce an equal or greater growth benefit to *Gunnera* under N limitation than exogenously supplied N [19]. However, the epiphytic cyanobacteria maintain high levels of gene expression for photosynthesis and C fixation, at least on the holobiont scale [20]. This indicates they may be primarily acquiring their energy and C photoautotrophically and may not require organic C inputs from the moss to maintain their N-fixation activity. It is thus possible that the feathermoss may not provide anything in return for the fixed-N products. This raises the question of whether this association is unidirectional or, alternatively, a symbiotic nutritional mutualism—defined here as a persistent physical association [21], with bidirectional exchange of nutritive chemicals or information [22], wherein each organism functions as both consumer and resource [23].

Aside from the potential organic C provided to the cyanobacteria, another possible benefit could be sulfur (S) provision. We recently examined the genomic, transcriptomic, proteomic, and exoproteomic features of *Nostoc* species that can and cannot colonize feathermosses [20]. Many genes involved in organic S transport and metabolism, in particular alkane and aliphatic sulfonates, were present in genomes of colonization-competent *Nostoc* strains and absent from the incompetent strain’s genome [20]. When expanded to include all facultative symbiotic *Nostoc* strains (11 total), gene families for organic S transport and metabolism were overrepresented [19]. Global gene and protein expression analysis demonstrated induction of many of these S-related genes during colonization with the feathermoss, as compared to growth alone [20]. Along with numerous copies of sulfonate transporters, a gene coding for a putative alkane sulfonate monooxygenase (AMSO, Npun_F5882) is present in all facultative symbiotic *Nostoc* genomes and absent from the incompetent strain [19]. This enzyme catalyzes conversion of alkane sulfonates to sulfite, which could then feed essential L-cysteine production [24]. Related gene families are expressed by a soybean root-nodulating bacterium, *Bradyrhizobium japonicum*, while in the plant host [25], indicating the potential importance of this gene family and organic S in N-fixing symbioses with plants. We hypothesize that this gene may have been an essential horizontal genetic acquisition that allowed for *Nostoc* colonization and establishment of feathermoss associations.

In order to better constrain the commodities essential to this putative mutualism, we set out to examine organic S and C transfer from the moss to the cyanobacteria and compare it to N transfer from the cyanobacteria to the moss. We define “transfer” here as referring to the translocation of a chemical compound, including both active and passive mechanisms, in keeping with other studies using high-resolution imaging secondary ion mass spectrometry (NanoSIMS) paired with stable isotope probing (nanoSIP) (e.g., [26]). We utilized an axenic line of feathermoss *P. schreberi* [27] and setup a controlled experimental system to initiate colonization between *P. schreberi* and *Nostoc* strains. Using a targeted gene knockout, we generated a completely segregated cyanobacterial mutant cell line and demonstrate the essential role of the *Nostoc punctiforme* sulfur metabolism gene AMSO in establishment of the symbiosis. We then conducted a follow-up experiment to detect and quantify transfer of N, C, and S between *P. schreberi* and a cyanobacterial epiphyte isolated from *P. schreberi, Nostoc* sp. Moss2 (referred to as *Nostoc* M2 hereafter). Applying NanoSIMS and nanoSIP, we demonstrate bilateral elemental transfer.

## Materials and methods

### Moss and cyanobacteria growth conditions

Axenic moss tissues of *P. schreberi* were used for all experiments [27]. Nitrogen fixation rates were measured on triplicate 1 mL samples from 2-week-old cultures placed in 10 mL chromatography vials equipped with rubber septa and measured by acetylene reduction assay ([28] and Supplementary Methods). For the stable isotope, labeling *P. schreberi* was grown on ^33^S substituted BCD agar media overlaid with filter paper for 56 days. For 18 days prior to experiment initiation ^33^S-labeled *P. schreberi* (~200 gametophytes) were incubated with ^13^CO_2_ (Supplementary Methods).

### Construction of cyanobacterial mutant

A plasmid to replace the putative alkanesulfonate monooxygenase (AMSO, Npun_F5882) was constructed as detailed in Supplementary Methods. Plasmids were introduced into *N. punctiforme* via conjugation with *E. coli* as described by [29]. Resulting *N. punctiforme* colonies were patched and screened for whether a double-recombination event had occurred to introduce the neomycin resistance cassette into the target gene using the upstream forward primer and the downstream reverse primer. Colonies that had the expected 3-kb band indicating double crossover into the target gene were selected for passaging to segregate the mutants. Segregation and confirmation of gene expression was done by RT-PCR (Supplementary Methods, Fig. S1), with the primers found in Supplementary Table S1.

### Colonization experimental design and infection rates

The colonization experiment was set up as described in [18]. Briefly, permeable polycarbonate membrane inserts with 8 µm pore size were used to allow attraction and colonization of the cyanobacteria to the moss, using 1/5 Bg11_0_ liquid growth media below the inserts. Four replicates of each treatment with different cyanobacterial strains were incubated in well plates. To assess rates of colonization, the inserts with the moss were blotted onto filter paper to remove excess media and free-living cyanobacteria, then placed into a 24-welled black plate (PerkinElmer, USA) and read daily on a Hidex sense microplate reader (Hidex, Turku, Finland). Readings were at 490 nm emission, 520 excitation nm to detect the phycobilin photosynthetic accessory pigments in cyanobacteria [30], which corresponds to the amount of cyanobacteria that has colonized the moss. As a control, 10 µM Fluorescein (Sigma Aldrich) was used in every run to ensure all values were corrected for during the experiment. Due to possible variable background autofluorescence from both the filters and the moss, positive colonization was determined by consistent increase in fluorescence over time (determined by a linear regression analysis).

### Triple stable isotope probing experiments

^33^S-labeled and ^13^C-labeled (“labeled”) and natural abundance control (“unlabeled”) *P. schreberi* gametophytes and unlabeled *Nostoc* M2 liquid culture were acclimated to 1/5 Bg11_0_ liquid media for 24 h, after which gametophytes and acclimated *Nostoc* M2 culture were primed for colonization by incubating together for 24 h under chemical contact [20]. Wells were incubated in sealed labeling chambers with ^13^CO_2_ or ^12^CO_2_ for labeled and unlabeled gametophytes, respectively, as described in Supplementary Methods. To allow initiation of colonization, primed gametophytes were rinsed with sterile unlabeled media to remove any excess label and incubated with primed unlabeled *Nostoc* M2 cultures for 6 h in unlabeled media and headspace. Colonized gametophytes (either labeled or unlabeled, ~10 per vial) were subsequently placed into five prepared sterilized 37.2 mL Wheaton vials with 3 mL of 1/5 BG11_0_ agar, sealed, and capped. A subset of labeled, primed, uncolonized gametophytes, and unlabeled *Nostoc* M2 cultures was also prepared, by incubating in 4% paraformaldehyde (PFA) for 1 h at room temperature, rinsing with sterile water to remove excess label, and placed together in a Wheaton vial, (referred to as “chemically killed” control treatment). ^15^N_2_ gas (99+ atom%, Cambridge Isotopes), for which contaminating ammonium [31] was removed by bubbling gas through 5% H_2_SO_4_ [32], was added to each vial at 58% ^15^N_2_ final concentration. For unlabeled control vials, an equivalent volume of unlabeled N_2_ was added. Sample vials were harvested at 24, 48 h and 12 days, gametophytes fixed with 4% PFA for 24 h at 4 °C, rinsed with three times with sterile water and stored in 50% EtOH at −20 °C. Unlabeled and chemically killed control treatments were harvested at 24 h. 10 mL of headspace gas was also collected for 24 h, unlabeled 24 h, and 12-day samples, and ^13^CO_2_/^12^CO_2_ data were collected using a Picarro G2201-i Cavity Ring Down Spectrometer (Picarro Inc., Santa Clara, CA, USA), with standard air samples run between each sample run.

For *Nostoc* M2 assimilation alone, *Nostoc* M2 liquid cultures were inoculated into 14 Wheaton vials, with 6 mL of BG11_0_ media, substituted with NaH^13^CO_3_^−^ (Sigma, 99+ atom%) and Na^33^SO_4_ (Sigma, 98+ atom%), and incubated for 21 days, with duplicate vials harvested at 0 and 6 h, and 1, 2, 7, 14, and 21 days. Additional NaH^13^CO_3_^−^ was injected every other day to prevent C limitation. Samples were preserved as described above.

### Sample preparation, mapping, and NanoSIMS stable isotope imaging

Gametophytes were screened for *Nostoc* M2 colonization using epifluorescence microscopy, with colonized phyllids removed and pressed onto a PELCO carbon conductive tab mounted on an SEM pin stub mount (Ted Pella, Redding CA). Phyllids from at least 3 moss gametophytes and at least 20 total cyanobacterial cells were analyzed for each time point. For control treatment gametophytes, uncolonized phyllids were mounted, and unlabeled fixed *Nostoc* M2 culture was pipetted directly on top of the phyllids and allowed to dry. SIMS imaging was performed with a CAMECA NanoSIMS 50 microprobe at Lawrence Livermore National Laboratory, as detailed in Supplementary Methods. Due to the high ^33^S enrichment of the moss tissue (mean ^33^S/^32^S of 0.96), redeposition of ^33^S ions onto the *Nostoc* M2 cells was a concern. Therefore, 1 × 1 µm^2^ spot analyses (32 × 32 pixels, 0.3 ms/pxl, 50 cycles) were used instead of image analysis for the cyanobacterial analyses (Fig. S2a). The cycle data for each spot analysis were inspected for significant increases in the ^33^S enrichment with depth, which would indicate intrusion into underlying highly ^33^S-enriched moss tissue (Fig. S2b, c); no such increases with depth were observed for these analyses. Sputtering of the surrounding region was limited while locating target cyanobacterial cells to minimize ion yield from the phyllids, and no pre-analysis sputtering was used because of the rapid rate of sample erosion with this small raster. This approach reduced ^33^S background from the phyllids to ~2.4%, as determined by fitting a linear regression model to the data for killed control cells on ^33^S- and ^13^C-labeled phyllids (Controls 1 and 2) and the unlabeled control (Fig. S3). The true ^33^S fraction for each cell (*f*_33S-cyano-est_) was estimated based on the slope of the regression in a mixing model, 0.024, and the measured ^33^S fraction for the phyllids (*f*_33S-moss-meas_) and cyanobacterial cells (*f*_33S-cyano-meas_):

1 $$0.024 \times f_{\rm{33S - moss - meas}} 	+ \left( {1 - 0.024} \right) \times f_{\rm{33S - cyano - est}} \\ 	 = f_{\rm{33S - cyano - meas}},$$

which was solved for *f*_33S-cyano-est_.

Image data were processed to extract ratio data from selected regions of interest using custom software (L’image, L. Nittler, Carnegie Institution of Washington), and the spot analysis isotopic ratios were calculated directly by the NanoSIMS software. The data are presented as atom percent excess (APE) [33, 34], as detailed in Supplementary Methods.

### Statistical analyses

All statistical analyses were performed using Prism Graphpad (Graphpad Software, San Diego CA). Values between treatment and control for each isotope were compared using a one-way ANOVA with a Dunnett’s post hoc test to compare each time point to the control (alpha: 0.05). For the ^15^N cyanobacteria results, a one-way ANOVA with a Tukey post hoc test was also conducted to compare between time points (alpha: 0.05). As part of our one-way ANOVA, we report *R*^2^ values, which quantify the strength of the relationship between group membership and the variable measured. Simple linear regression analyses were performed on colonization data to determine whether a slope deviated significantly from 0 (*p* < 0.01). Pearson correlation analyses were performed comparing mean cyanobacterial ^15^N APE with mean ^13^C APE and ^33^S APE, respectively, at each time point (two tailed, alpha of 0.05). For the *Nostoc* M2 growth alone, a Pearson correlation analysis (two tailed, alpha of 0.05) and simple linear regression were performed on all cells analyzed to test correlation between ^13^C and ^33^S uptake over time.

## Results

### Role of Npun_5882, a putative AMSO, in moss colonization

**Fig. 1:**
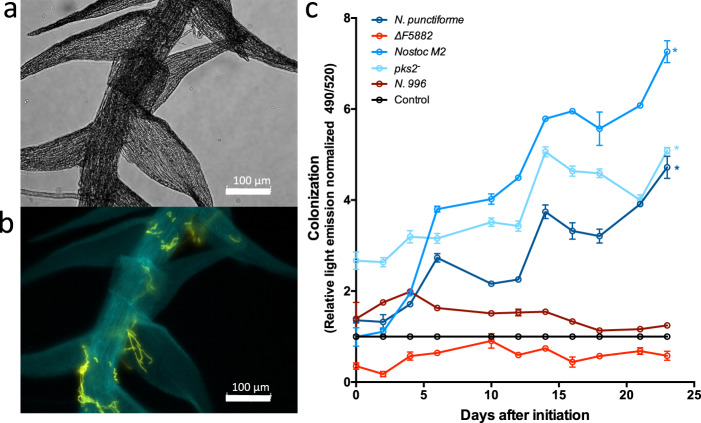
Assessment of colonization of *P. schreberi* by *Nostoc strains*. **a**, **b** Micrographs of *P. schreberi* gametophyte with *Nostoc* M2 colonization. Brightfield image (**a**) shows moss gametophyte, and fluorescent image (**b**) shows *Nostoc* M2 filaments (yellow) on gametophyte (cyan). **c** Colonization of different cyanobacterial strains on the axenic *P. schreberi* line, including: wild-type *Nostoc punctiforme*, the model strain, and *Nostoc* M2 colonization-competent field isolate, ΔF5882, our AMSO gene mutant, *pks2*^*−*^ control mutant, *Nostoc* sp. N996 colonization-incompetent control. “Control” is *P. schreberi* incubated without cyanobacteria. Error bars represent one standard deviation between four biological replicates. “*” indicates significant linear regression slope (*p* < 0.01) >0.

**Fig. 2:**
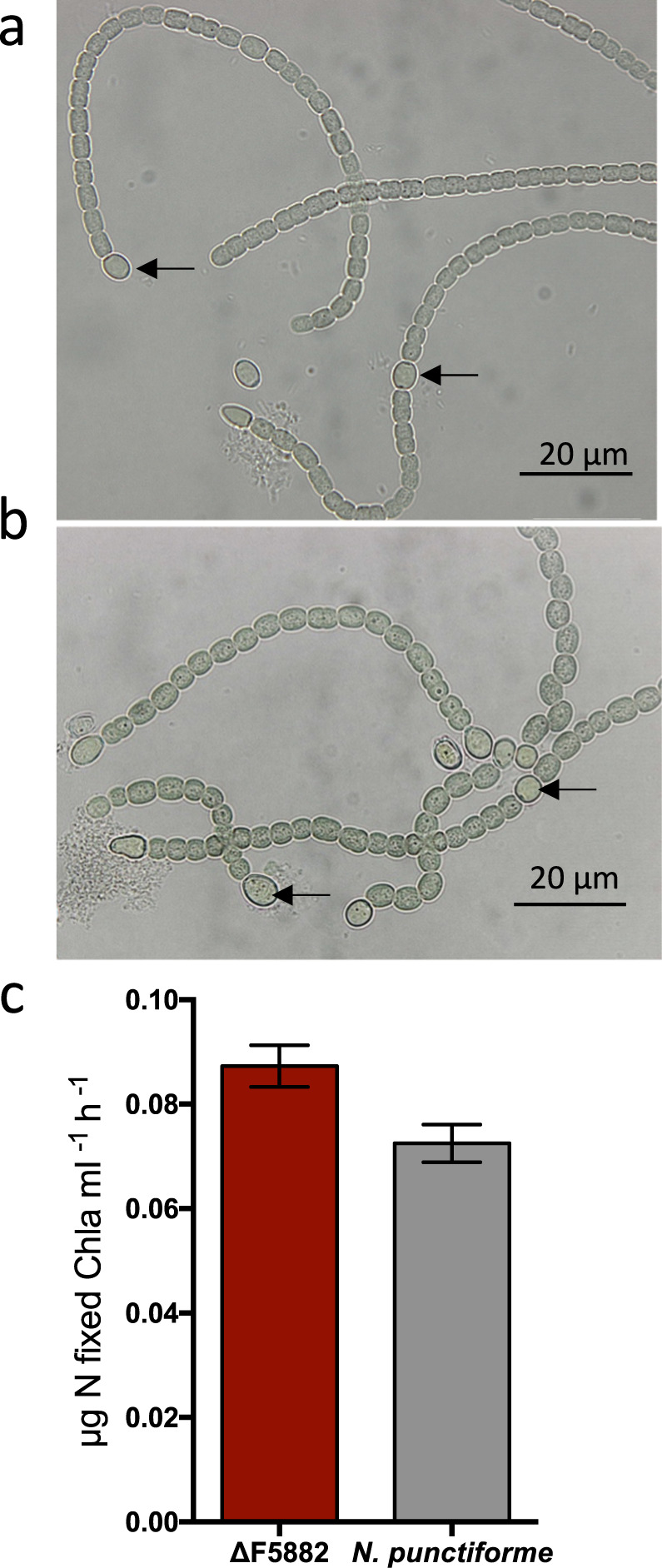
F5882 nitrogen fixation capabilities compared to wild-type *N. punctiforme*. Light microscopy of *N. punctiforme* (**a**) and mutant ΔF5882 (**b**), showing heterocyst formation. Black arrows point out representative heterocysts. **c** Nitrogen fixation rates using acetylene reduction assay in wild type and mutant, expressed as extracted chlorophyll *a* ml^−1^ culture. Error bars represent one standard deviation between triplicates.

To investigate whether Npun_5882 may play a role in this symbiosis, we generated a completely segregated mutant (*N. punctiforme* ΔF5882::Neo^R^, referred to as ΔF5882 hereafter) deficient in the FMNH(2)-dependent alkane sulfonate monooxygenase (“AMSO,” Npun_F5882) gene in the *N. punctiforme* PCC 73102 background. We utilized the newly developed axenic *P. schreberi* line [27], allowing for rapid, replicated, and repeatable colonization assays (Fig. 1a, b). ΔF5882 was deficient in colonization of *P. schreberi* compared to: wild type, *Nostoc* M2, and another mutant *N. punctiforme* strain, *pks2*^*−*^ [35] (Fig. 1c). Linear regression analyses indicated significant (*p* < 0.01) positive slopes for wild type, *Nostoc* M2, and *pks2*^*−*^ (Fig. 1c). By contrast, the slope for ΔF5882 was not significantly different from 0 (*p* = 0.1976), and the known symbiosis-incompetent strain, *Nostoc* sp. CALU 996 (N. 996) [20], had a slightly negative slope (*p* = 0.010). There were some differences in the positive slopes between colonization-competent strains (wild type, *Nostoc* M2, and *pks2*^−^), but since the experiment was not designed to compare rates of colonization, we cannot rule out confounding factors. We also verified the capability of ΔF5882 for N-fixation and heterocyst formation (Fig. 2). This indicates that the inability of ΔF5882 to colonize is independent from these other necessary functions. N-fixation was slightly higher in the mutant, but a time-resolved analysis would be required to confirm this. These results establish the AMSO gene in *N. punctiforme* as essential for colonization, and, in turn, that cyanobacterial catabolism of alkane sulfonates is linked to colonization.

### NanoSIP and transfer of N, C, and S between *P. schreberi* and *Nostoc* M2 over time

**Fig. 3:**
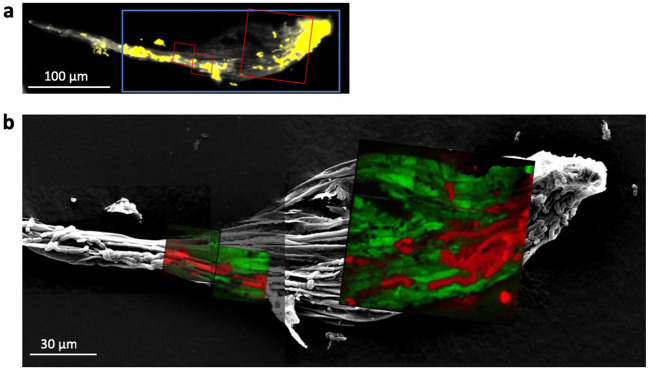
Representative images used for NanoSIP sample visualization. Representative moss phyllid from 12-day incubation, imaged with epifluorescence microscopy (**a**), scanning electron microscopy (**b**, gray), and NanoSIMS merged ratio images (**b**). Green indicates ^33^S/^32^S ratio and red indicates ^15^N^12^C/^14^N^12^C ratio.

Using stable isotope probing combined with NanoSIMS (“nanoSIP,” [33]), we examined ^15^N, ^13^C, and ^33^S label assimilation into single *Nostoc* M2 cells colonizing *P. schreberi* phyllids, as well as underlying *P. schreberi* tissue. The *Nostoc* M2 strain was isolated from *P. schreberi* and chosen for its ecological relevance. ^15^N_2_ (gas) was added at the initiation of the 12-day incubation, following the 6 h colonization incubation, allowing for quantification of N-fixation by the cyanobacterium and transfer of fixed-N products to the moss. Using correlated fluorescence microscopy and SEM, we identified locations on phyllid surfaces with *Nostoc* M2 colonization (Figs. 3 and S4) for subsequent nanoSIP analyses at 24, 48 h and 12 days after experiment initiation. In this experimental setup, only the cyanobacterium is capable of N-fixation, so enrichment of ^15^N in the moss indicates N fixed by the cyanobacterium is transferred to the moss. As the moss is pre-labeled with ^13^C and ^33^S, enrichment of the cyanobacterial cells in these isotopes indicates reverse transfer, though as discussed below, the C transfer requires some estimates of respiration versus photosynthesis by the moss.

**Fig. 4:**
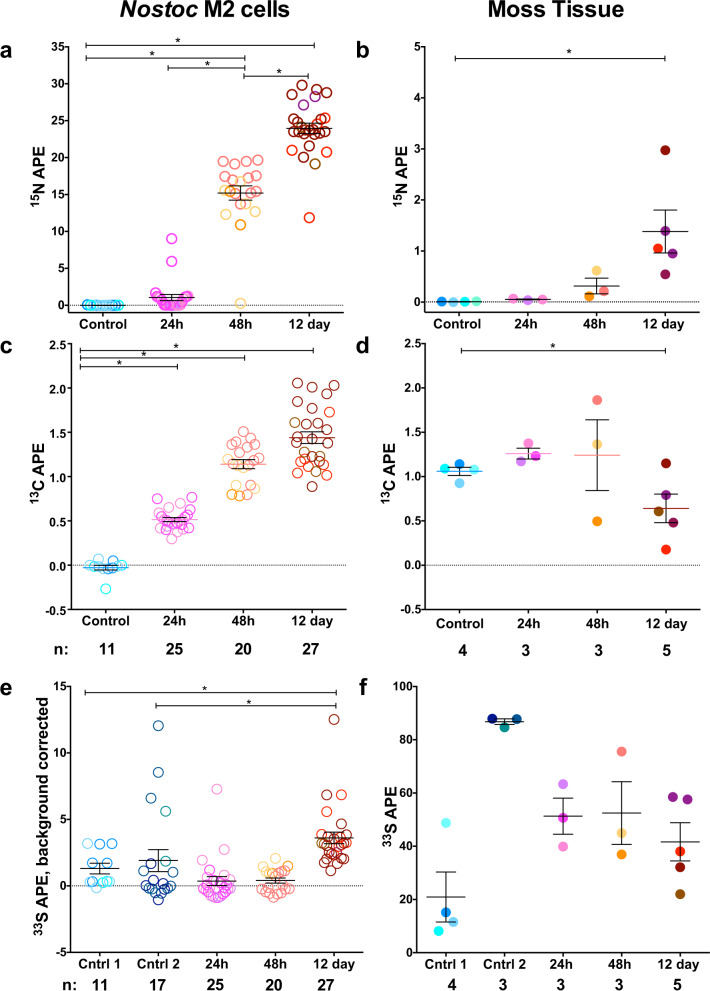
NanoSIP single cell and moss tissue enrichment for three stable isotopes over time. NanoSIMS-derived ^15^N APE (**a**, **b**), ^13^C APE (**c**, **d**), and ^33^S APE (**e**, **f**) values for single *Nostoc* M2 cells (left) and moss tissue (right), at three successive time points following colonization. “Control” indicates treatment wherein both moss and *Nostoc* M2 were chemically killed before incubation. Brackets and star indicates significant (*p* < 0.05) difference from control. (**e**) Ratios have been background corrected for contrition of the highly labeled moss tissue. Different color hues in each column represent different moss phyllids (**b**, **d**, **f**) or single cells (**a**, **c**, **e**) originating from different phyllids. Each point represents a single cell or tissue measurement, and error bars represent one standard error of the mean (*n* values on graph).

As anticipated based on previous results [18], N transfer from the cyanobacterium to the moss was observed (Fig. 4a, b). This transfer was evident in some of the moss tissue analyzed at 24 and 48 h following initiation of ^15^N_2_ labeling, with significant increases in ^15^N enrichment (APE) in all gametophyte moss tissue analyzed by 12 days, relative to the control (Fig. 4a, b, F(4, 13) = 5.512, *p* = 0.0081, *R*^2^ = 0.63). In the cyanobacterium, N-fixation was detectable in some *Nostoc* M2 cells at 24 h. By 48 h there was a significant shift in the mean ratio relative to the control (F(4, 83) = 238.8, *p* < 0.0001, *R*^2^ = 0.92). ^15^N enrichment increased through 12 days, with the means between 24 and 48 h significantly different, as well as between 48 h and 12 days (Figs. 4a and S5), indicating that the cyanobacterium is fixing N constantly while colonizing the moss. The increase between 24 and 48 h is greater (14.15 ± 0.937 mean increase) than the increase between 48 h and 12 days (8.75 ± 0.920 mean increase) (Fig. S5), indicating a possible reduction of, but still continued, net N-fixation after 48 h.

The ^13^C enrichment data demonstrate that moss-derived C is transferred to the cyanobacterium. The moss was ^13^C labeled prior to the initiation of colonization and subsequent incubation to 0.92–1.14 ^13^C APE (Fig. 4). The cyanobacterium was statistically significantly enriched in ^13^C by 24 h, relative to the control, and was higher at 48 h and 12 days (Fig. 4c, F(4, 83) = 119.7, *p* < 0.0001, *R*^2^ = 0.85). The moss ^13^C enrichment was not significantly different from the control at the 24 and 48 h time points, but by 12 days, the mean moss enrichment was lower, due to dilution of the label from moss fixation of the natural abundance CO_2_ in the incubation vial (Fig. 4d, F(4, 13) = 7.041, *p* = 0.003, *R*^2^ = 0.68). Since the cyanobacterium is also fixing CO_2_, some of the ^13^C cyanobacterial enrichment could be coming from ^13^CO_2_ respired by the moss. Consequently, we measured ^13^CO_2_ in the headspace in order to constrain the input C sources (Supplementary Table S2). The ^13^C APE of the CO_2_ was 0.33 ± 0.01^13^C APE at 24 h, which is lower than the mean cyanobacterial enrichment at that time (0.51 ± 0.02, Fig. 4c), indicating that at least some of the C originated from moss-derived organic C. By 12 days the ^13^C APE of CO_2_ in the headspace had decreased to match the no-isotope control (−0.023 ± 0.009 and −0.016 ± 0.010, respectively), while the mean enrichment of the cyanobacterium (1.44 ± 0.34) was higher than the earlier time point and the bulk moss tissue.

**Fig. 5:**
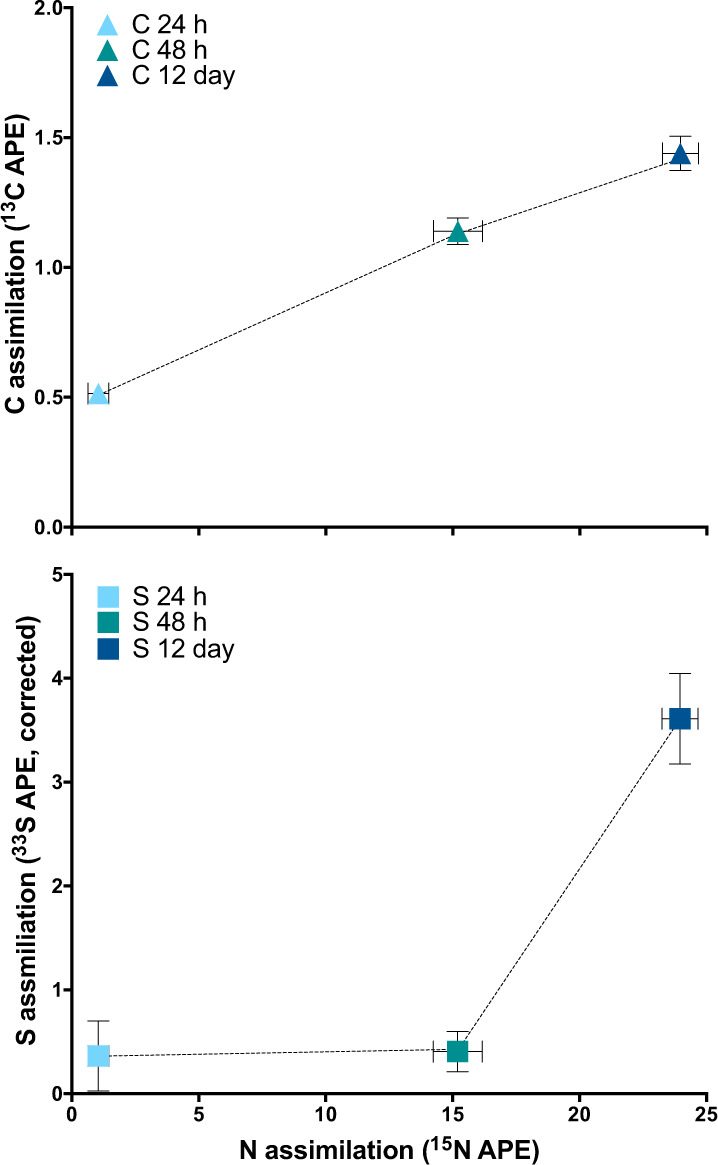
Comparing dynamics of cyanobacterial carbon and sulfur uptake  relative to nitrogen fixation. Mean APE of *Nostoc* M2 for each of the stable isotopes. Each point represents the mean APE of 20–27 single cell analyses from the three time points for either ^33^S and ^15^N (top) or ^13^C and ^15^N (bottom). Error bars represent one standard error of the mean. Pearson correlation for ^13^C APE was significant (*r* = 0.998, *p* = 0.042), and not for ^33^S APE (*r* = 0.798, *p* = 0.41).

The ^33^S enrichment data demonstrate S transfer from the moss to the cyanobacterium, at slower rate than C. The moss gametophytes were cultured on media with ^33^S-enriched sodium sulfate as the only S source and were highly enriched in ^33^S prior to colonization and incubation (Fig. 4). This high level of moss labeling contributed to background ^33^S signal for our analyses of control cyanobacterial cells, which were unlabeled and located on labeled moss tissue. Consequently, two moss tissue controls with differing levels of ^33^S enrichment (Fig. 4f) were used to calculate ^33^S background as a function of moss enrichment and correct the cyanobacterial cell ^33^S/^32^S measurements (Fig. S3). In contrast with the pattern observed for ^13^C, the cyanobacterial cells were not enriched in ^33^S at 24 and 48 h. However, the mean at 12 days did significantly increase compared to each of the controls (Fig. 4e, F(4, 83) = 16.71, *p* < 0.0001, *R*^2^ = 0.45; F(4, 91) = 9.579, *p* < 0.0001, *R*^2^ = 0.30). Total mean assimilation of ^33^S in the cyanobacterial cells by 12 days was 3.9 ± 2.2 APE compared to the average moss tissue at 12 days of 41.6 ± 7.2 APE. Furthermore, mean C transfer correlated linearly with mean N-fixation over time, whereas S transfer did not significantly increase until after 48 h (Fig. 5).

To put these results in context, we conducted an additional experiment to examine direct sulfate assimilation by *Nostoc* M2 cultured photoautotrophically in isolation with Na_2_^33^SO_4_ (99 at%) and ^13^C-bicarbonate (99 at%). This resulted in a 7 fold higher rate of sulfur assimilation relative to the rate of sulfur assimilation from the moss when grown together, assuming that the S provided by teh moss had the average moss 33S enrichment (Fig. S6a). In addition, in these cultures, we found sulfate assimilation correlated linearly with C fixation (*r* = 0.89, *R*^2^ = 0.79, *p* < 0.0001) (Fig. S6b), indicating S is required for growth in a consistent stoichiometry with C.

## Discussion

We set out to characterize the nature of nutrient exchange between epiphytically associated cyanobacteria and *P. schreberi*, which was unresolved. Using a combination of targeted mutagenesis and nanoSIP, we show that (1) a previously unrecognized gene family is essential for the colonization and (2) there is bidirectional mutualistic elemental transfer between the moss and cyanobacterium.

Our genetic manipulation results demonstrate that a gene coding for a putative AMSO is essential for feathermoss colonization (Fig. 1). There is evidence that utilization of organic S sources may be important to other diazotrophic symbiont taxa; *Bradyrhizobium japonicum*, a soybean root-nodulating bacterium, was found to express a suite of genes under control of a N-fixation regulator when grown with sulfonates as the sole S source and also expressed these sulfonate utilization genes, including AMSO, while in the symbiotic root nodules [25]. In addition, like in *Nostoc*, these genes are predicted to be part of an important host adaptation [36]. Our mutant, *N. punctiforme* ΔF5882, was capable of N-fixation and differentiation of heterocysts, two phenotypic adaptations previously known to be important for stable and efficient symbiosis [37, 38]. This suggests that the deficiency in colonization has to do with utilization or detection of organic alkane sulfonate compounds and is separate from the ability to provide N to the moss (which has already been shown to be important for colonization [18]). These S compounds likely originate from the moss, since *N. punctiforme* does not appear to have the required biosynthetic pathways. However, based on the global gene expression profiles from Warshan et al. [20], it appears that cyanobacteria colonizing mosses are not S limited. While AMSO does not appear to be significantly upregulated when in contact with the moss, aliphatic sulfonate transporters in a predicted operon with AMSO are upregulated, whereas sulfate transporters are all downregulated ([20] and Supplementary Table S3). Given the lack of S limitation signatures, this suggests the possibility of an additional or alternative role from nutrition for the alkane sulfonates.

Our nanoSIP results also point to some differences in S transfer compared to C and N. S transfer by nanoSIP is only detectable after 12 days of colonization, indicating that this process is slower and lower than C transfer. Furthermore, whereas moss-derived C assimilation correlated with N-fixation over time, moss-derived S assimilation did not (Fig. 5). This is unlikely to be solely the result of a low S requirement, or low detection limits, since the *Nostoc* M2 culture clearly exhibited a rapid increase in ^33^S/^32^S ratios when the cyanobacterium in isolation was provided with ^33^S-enriched sulfate. Even considering that growth rates are likely to be lower when in symbiosis, as has been shown in the endophytic symbioses [39], this level of S transfer, while significant, is unlikely enough to satisfy growth requirements.

Taken together, these results indicate that the organic S commodity provided by the moss host may play a different role than solely nutrient provision. One possibility is that the organic S transfer observed is primarily the exchange of a communication molecule. Such molecules would be expected to accumulate in lower levels. Communication with the moss is likely important for several stages of the association. For example, in the endophytic cyanobacterial–plant symbioses, hosts such as the hornwort *Anthoceros*, liverwort *Blasia*, angiosperm *Gunnera*, and cycads, are known to influence motile hormogonia development through production of inducing factors (HIF). In addition, hormogonia-repressing factors in *Anthoceros*, heterocyst induction factors in *Anthoceros* and *Gunnera*, and cell division repression in *Anthoceros* and cycads have been detected (for review see [40]). Feathermosses also make HIF, although some species that induce hormogonia do not form associations with *Nostoc*, indicating there are additional mechanisms required for colonization [18]. While an HIF isolated from cycad roots has been recently characterized [41], most of these molecules remain uncharacterized chemically and could include S groups. Hydrogen sulfide is a known plant signaling molecule [42], and S-containing molecules can play a role in redox-related signaling [43]. Furthermore, in nodule-forming diazotrophic symbioses, Nod factors (produced by the symbiont rhizobia to induce host nodulation) can be sulfated and may play a role in host specificity [44]. However, as far as we are aware, sulfonated plant hormones have not been described, so this could be a novel plant signaling molecule. If the symbiosis-competent *Nostoc* can assimilate these specific compounds to the exclusion of other potential colonizing bacteria, as reflected by their genomic content [19], the moss host may be using S metabolites to regulate microbial colonization.

Our results further indicate continuous organic C transfer from the moss to the cyanobacterium, correlated with cyanobacterial N-fixation, which was unexpected, given the global gene and protein expression profiling [20]. We cannot rule out, and indeed believe, that the cyanobacterium is also fixing CO_2_. However, in this closed system with only one input of enriched ^13^C (the moss tissue), the cyanobacteria cell enrichment data and the headspace analysis indicate that they are also receiving organic C from the moss. Specifically, in the cyanobacterial cells we see an increase in ^13^C enrichment over time, above the ^13^C enrichment in the headspace. By contrast, in the moss tissue, whose only C input is CO_2_ fixation, we see a decrease in enrichment, even though the moss is likely to have grown less than the cyanobacteria in this 12-day study. By 12 days, the cyanobacterial ^13^C enrichment exceeded the bulk moss tissue enrichment, indicating that the cyanobacteria have obtained recently fixed moss C. While organic C provision by the host is in line with other cyanobacterial–plant associations such as cycads and *Gunnera* [15], global expression data had revealed that photosynthesis and C fixation gene transcripts were still among the top most abundant transcripts during chemical and physical association with the moss, despite some slight downregulation relative to growth in isolation [20]. This was distinct from other associations, where the cyanobacteria dramatically decrease photosynthesis and C fixation gene expression [17, 37]. Simultaneous organic C assimilation and CO_2_-fixation has been shown for other cyanobacterial groups, which supplement their photoautotrophic C with organic sources [45, 46]. It is also possible that despite high transcript and protein levels, RubisCO activity is low *in planta*, as shown in the *Azolla-*cyanobacteria symbiosis [47, 48]. A final possibility is that *Nostoc* M2 is segregating carbon metabolic activities between heterocysts assimilating and respiring organic C, and vegetative cells continuing to fix CO_2_, which could be explored by more targeted NanoSIP experiments. Regardless, our results indicate that organic C is one of the commodities provided by the moss, likely to fuel N-fixation.

This work sheds new light on still-open questions regarding how the symbiosis is maintained. One mechanism to maintain cooperation in a complex network of interactors is to employ bidirectional control of nutrient transfer, as seen in mycorrhizal symbioses [49]. It is unclear whether this is the case for the feathermoss–cyanobacterium symbiosis. Bidirectional control requires some degree of exchange exclusivity, which in this epiphytic association is a potential challenge, since the exuded molecules from either moss or cyanobacteria might be available to opportunistic microbes, or “cheater” partners. From microscopic observations, there does not appear to be a septum or shared membrane between the partners, so it is expected that transfer is indirect, through exudation. It is known that these filamentous cyanobacteria have dedicated transporters for transferring substrates such as glutamine and sugars between cells on a filament [50], but it is also hypothesized that there may be passive diffusion between cells [51]. Exclusivity could be maintained through compound specificity and signaling. Identifying the moss-provided C and S compounds, their metabolic costs (e.g., metabolic by-products or specialized compounds), and their general bioavailability would help resolve this. There is also the possibility that other nutrients, such as Mo and P [52], could also be transferred along with the C and play a role in maintaining cooperation, which could be resolved by characterizing the compounds transferred. Another possibility is that non-nutritional benefits like protection could play a role in maintaining cooperation, as suggested for the *Sphagnum-*cyanobacteria association [53]. Future work to determine the identity of transferred molecules and regulation of their production will provide insight into the selective forces governing stability of the mutualism.

Collectively, these results demonstrate the multifaceted nature of metabolic transfer between feathermoss and cyanobacteria, establishing a mutualistic symbiosis and suggesting roles for exchange of these nutrients in initiation and maintenance of the symbiosis in natural systems. Continuous N transfer to the moss likely drives moss benefits, as expected. Carbon nutrition may fuel cyanobacterial N-fixation, while transfer of S may play a role in initiation with appropriate partners. These results also provide a novel avenue for the role of S in any plant–microbial symbiosis, one distinct from C and N nutrition, providing new directions for future work into chemical signaling between the two partners including chemical identification of the transferred signaling and nutritional compounds.

## Supplementary information

Supplementary Information

## Data Availability

The data that support the findings of this study are available from the corresponding author upon reasonable request.

## References

[1] Goodale CL, Apps MJ, Birdsey RA, Field CB, Heath LS, Houghton RA (2002). Forest carbon sinks in the Northern Hemisphere. Ecol Appl.

[2] Anderson J (1991). The effects of climate change on decomposition processes in grassland and coniferous forests. Ecol Appl.

[3] Turetsky MR, Bond‐Lamberty B, Euskirchen E, Talbot J, Frolking S, McGuire AD (2012). The resilience and functional role of moss in boreal and arctic ecosystems. N Phytol.

[4] DeLuca TH, Zackrisson O, Gundale MJ, Nilsson M-C (2008). Ecosystem feedbacks and nitrogen fixation in boreal forests. Science.

[5] Nilsson M-C, Wardle DA (2005). Understory vegetation as a forest ecosystem driver: evidence from the northern Swedish boreal forest. Front Ecol Environ.

[6] Rousk K, Jones D, DeLuca T (2013). Moss-cyanobacteria associations as biogenic sources of nitrogen in boreal forest ecosystems. Front Microbiol.

[7] Carleton T, Read D (1991). Ectomycorrhizas and nutrient transfer in conifer–feather moss ecosystems. Can J Bot.

[8] Gundale MJ, Nilsson M, Bansal S, Jäderlund A (2012). The interactive effects of temperature and light on biological nitrogen fixation in boreal forests. N Phytol.

[9] Gundale MJ, Wardle DA, Nilsson M-C (2012). The effect of altered macroclimate on N-fixation by boreal feather mosses. Biol Lett.

[10] Jackson BG, Martin P, Nilsson M-C, Wardle DA (2011). Response of feather moss associated N2 fixation and litter decomposition to variations in simulated rainfall intensity and frequency. Oikos.

[11] Jean M-E, Cassar N, Setzer C, Bellenger J-P (2012). Short-term N2 fixation kinetics in a moss-associated cyanobacteria. Environ Sci Technol.

[12] Sorensen PL, Lett S, Michelsen A (2012). Moss-specific changes in nitrogen fixation following two decades of warming, shading, and fertilizer addition. Plant Ecol.

[13] Warshan D, Bay G, Nahar N, Wardle DA, Nilsson MC, Rasmussen U (2016). Seasonal variation in nifH abundance and expression of cyanobacterial communities associated with boreal feather mosses. ISME J.

[14] Rai AN, Soderback E, Bergman B (2000). Tansley review No. 116 cyanobacterium–plant symbioses. N Phytol.

[15] Meeks JC, Pawlowski K (2009). Physiological adaptations in nitrogen-fixing *Nostoc*–plant symbiotic associations. Prokaryotic symbionts in plants.

[16] Steinberg NA, Meeks JC (1991). Physiological sources of reductant for nitrogen fixation activity in *Nostoc* sp. strain UCD 7801 in symbiotic association with *Anthoceros punctatus*. J Bacteriol.

[17] Khamar HJ, Breathwaite EK, Prasse CE, Fraley ER, Secor CR, Chibane FL (2010). Multiple roles of soluble sugars in the establishment of *Gunnera-Nostoc* endosymbiosis. Plant Physiol.

[18] Bay G, Nahar N, Oubre M, Whitehouse MJ, Wardle DA, Zackrisson O (2013). Boreal feather mosses secrete chemical signals to gain nitrogen. N Phytol.

[19] Warshan D, Liaimer A, Pederson E, Kim S-Y, Shapiro N, Woyke T (2018). Genomic changes associated with the evolutionary transitions of *Nostoc* to a plant symbiont. Mol Biol Evol.

[20] Warshan D, Espinoza JL, Stuart RK, Richter RA, Kim S-Y, Shapiro N (2017). Feathermoss and epiphytic *Nostoc* cooperate differently: expanding the spectrum of plant-cyanobacteria symbiosis. ISME J.

[21] Douglas AE. The symbiotic habit. Princeton, NJ: Princeton University Press; 2010.

[22] Bronstein JL (2015). Mutualism.

[23] Holland JN, Ness JH, Boyle A, Bronstein JL. Mutualisms as consumer-resource interactions. Ecology of predator–prey interactions. Oxford, UK: Oxford University Press; 2005. p. 17–33.

[24] van der Ploeg JR, Eichhorn E, Leisinger T (2001). Sulfonate-sulfur metabolism and its regulation in *Escherichia coli*. Arch Microbiol.

[25] Sugawara M, Shah GR, Sadowsky MJ, Paliy O, Speck J, Vail AW (2011). Expression and functional roles of *Bradyrhizobium japonicum* genes involved in the utilization of inorganic and organic sulfur compounds in free-living and symbiotic conditions. Mol Plant Microbe Interact.

[26] Musat N, Foster R, Vagner T, Adam B, Kuypers MMM (2012). Detecting metabolic activities in single cells, with emphasis on nanoSIMS. FEMS Microbiol Rev.

[27] Pederson ERA, Warshan D, Rasmussen U (2019). Genome sequencing of *Pleurozium schreberi:* the assembled and annotated draft genome of a pleurocarpous feather moss. G3: Genes, Genomes, Genetics.

[28] Hardy RW, Holsten R, Jackson E, Burns R (1968). The acetylene-ethylene assay for N2 fixation: laboratory and field evaluation. Plant Physiol.

[29] Khayatan B, Bains DK, Cheng MH, Cho YW, Huynh J, Kim R (2017). A putative O-linked β-N-acetylglucosamine transferase is essential for hormogonium development and motility in the filamentous cyanobacterium *Nostoc punctiforme*. J Bacteriol.

[30] Falkowski PG, Raven JA. Aquatic photosynthesis. Princeton, NJ: Princeton University Press; 2013.

[31] Dabundo R, Lehmann MF, Treibergs L, Tobias CR, Altabet MA, Moisander PH (2014). The contamination of commercial 15N2 gas stocks with 15N–labeled nitrate and ammonium and consequences for nitrogen fixation measurements. PloS ONE.

[32] Ndegwa PM, Vaddella VK, Hristov AN, Joo HS (2009). Measuring concentrations of ammonia in ambient air or exhaust air stream using acid traps. J Environ Qual.

[33] Pett-Ridge J, Weber PK. NanoSIP: NanoSIMS applications for microbial biology. Microbial systems biology. Totowa, NJ: Humana Press; 2012. p. 375–408. 10.1007/978-1-61779-827-6_13 22639220

[34] Popa R, Weber PK, Pett-Ridge J, Finzi JA, Fallon SJ, Hutcheon ID (2007). Carbon and nitrogen fixation and metabolite exchange in and between individual cells of *Anabaena oscillarioides*. ISME J.

[35] Liaimer A, Helfrich EJN, Hinrichs K, Guljamow A, Ishida K, Hertweck C (2015). Nostopeptolide plays a governing role during cellular differentiation of the symbiotic cyanobacterium *Nostoc punctiforme*. Proc Natl Acad Sci USA.

[36] Koch M, Delmotte N, Rehrauer H, Vorholt JA, Pessi G, Hennecke H (2010). Rhizobial adaptation to hosts, a new facet in the legume root-nodule symbiosis. Mol Plant Microbe Interact.

[37] Meeks JC, Elhai J (2002). Regulation of cellular differentiation in filamentous cyanobacteria in free-living and plant-associated symbiotic growth states. Microbiol Mol Biol Rev.

[38] Wong FC, Meeks JC (2002). Establishment of a functional symbiosis between the cyanobacterium *Nostoc punctiforme* and the bryophyte *Anthoceros punctatus* requires genes involved in nitrogen control and initiation of heterocyst differentiation. Microbiology.

[39] Hill DJ (1989). The control of the cell cycle in microbial symbionts. N Phytol.

[40] Adams DG, Duggan PS. Signalling in cyanobacteria–plant symbioses. Signaling and communication in plant symbiosis. New York City, NY: Springer; 2012. p. 93–121.

[41] Hashidoko Y, Nishizuka H, Tanaka M, Murata K, Murai Y, Hashimoto M (2019). Isolation and characterization of 1-palmitoyl-2-linoleoyl-sn-glycerol as a hormogonium-inducing factor (HIF) from the coralloid roots of Cycas revoluta (Cycadaceae). Sci Rep.

[42] Calderwood A, Kopriva S (2014). Hydrogen sulfide in plants: from dissipation of excess sulfur to signaling molecule. Nitric Oxide.

[43] Koppenol WH, Bounds PL (2017). Signaling by sulfur-containing molecules. Quantitative aspects. Arch Biochem Biophys.

[44] Miller JB, Oldroyd GE. The role of diffusible signals in the establishment of rhizobial and mycorrhizal symbioses. Signaling and communication in plant symbiosis. New York City, NY: Springer; 2012. p. 1–30.

[45] Duhamel S, Van Wambeke F, Lefevre D, Benavides M, Bonnet S (2018). Mixotrophic metabolism by natural communities of unicellular cyanobacteria in the western tropical South Pacific Ocean. Pacific Ocean. Environ Microbiol.

[46] Stuart RK, Mayali X, Lee JZ, Everroad RC, Hwang M, Bebout BM (2016). Cyanobacterial reuse of extracellular organic carbon in microbial mats. ISME J.

[47] Kaplan D, Peters GA (1988). Interaction of carbon metabolism in the *Azolla-Anabaena* symbiosis. Symbiosis.

[48] Ray TB, Mayne BC, Toia RE, Peters GA (1979). *Azolla-Anabaena* relationship: VIII. Photosynthetic characterization of the association and individual partners. Plant Physiol.

[49] Kiers ET, Duhamel M, Beesetty Y, Mensah JA, Franken O, Verbruggen E (2011). Reciprocal rewards stabilize cooperation in the mycorrhizal symbiosis. Science.

[50] Nürnberg DJ, Mariscal V, Bornikoel J, Nieves-Morión M, Krauß N, Herrero A (2015). Intercellular diffusion of a fluorescent sucrose analog via the septal junctions in a filamentous cyanobacterium. mBio.

[51] Mullineaux CW, Mariscal V, Nenninger A, Khanum H, Herrero A, Flores E (2008). Mechanism of intercellular molecular exchange in heterocyst-forming cyanobacteria. EMBO J.

[52] Rousk K, Degboe J, Michelsen A, Bradley R, Bellenger JP (2017). Molybdenum and phosphorus limitation of moss‐associated nitrogen fixation in boreal ecosystems. N Phytol.

[53] Solheim B, Zielke M, Rai AN, Bergman B, Rasmussen U (2002). Associations between cyanobacteria and mosses. Cyanobacteria in symbiosis.

