# Supplementary material for: Bidirectional C and N transfer and a potential role for sulfur in an epiphytic diazotrophic mutualism
Source: ISME J. 2020 Aug 19;14(12):3068–78. doi: 10.1038/s41396-020-00738-4 (PMC7784912; doi:10.1038/s41396-020-00738-4)
Supplement: Supplementary file 1 — Supplementary Information [file 41396_2020_738_MOESM1_ESM.pdf]

Supplementary Information for:

Bidirectional exchange and a novel role for sulfur in a cyanobacteria-moss diazotrophic mutualism

Rhona K. Stuart[1*], Eric R.A. Pederson[2], Philip D. Weyman[3+], Peter K. Weber[1], Ulla Rassmussen[2], and Christopher L. Dupont[3*]

[1]Physical and Life Sciences Directorate, Lawrence Livermore National Laboratory, Livermore CA, USA

[2]Department of Ecology, Environment and Plant Sciences, Stockholm University, 106 91, Stockholm, Sweden

[3]J. Craig Venter Institute, La Jolla, CA 92037

[+]Current address: Zymergen Inc., Emeryville CA

Correspondence addresses: stuart25@llnl.gov, cdupont@jcvi.org

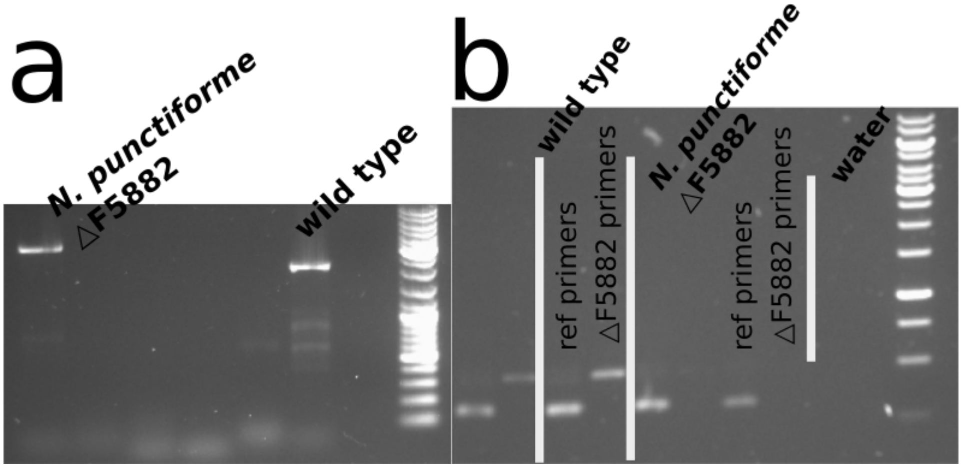

Supplementary Figure S1 Segregation of ΔF5882 was tested by PCR using primers gEP11 and gEP12 shown in (a). Lanes show *N. punctiforme* wild-type genomic DNA (WT) and ΔF5882 genomic DNA. The size difference between the mutant and wild type is clearly seen and expected as a result of the mutation. RT-PCR gene expression mutant confirmation (b) using primers rEP29 and rEP30 from cDNA synthesised from 0.5 μg total RNA extracted from the ΔF5882 mutant and wild type *N. punctiforme*. Lanes show wild-type cDNA (WT) and ΔF5882 DNA. Primers sequences utilized are found in Supplementary Table S1.

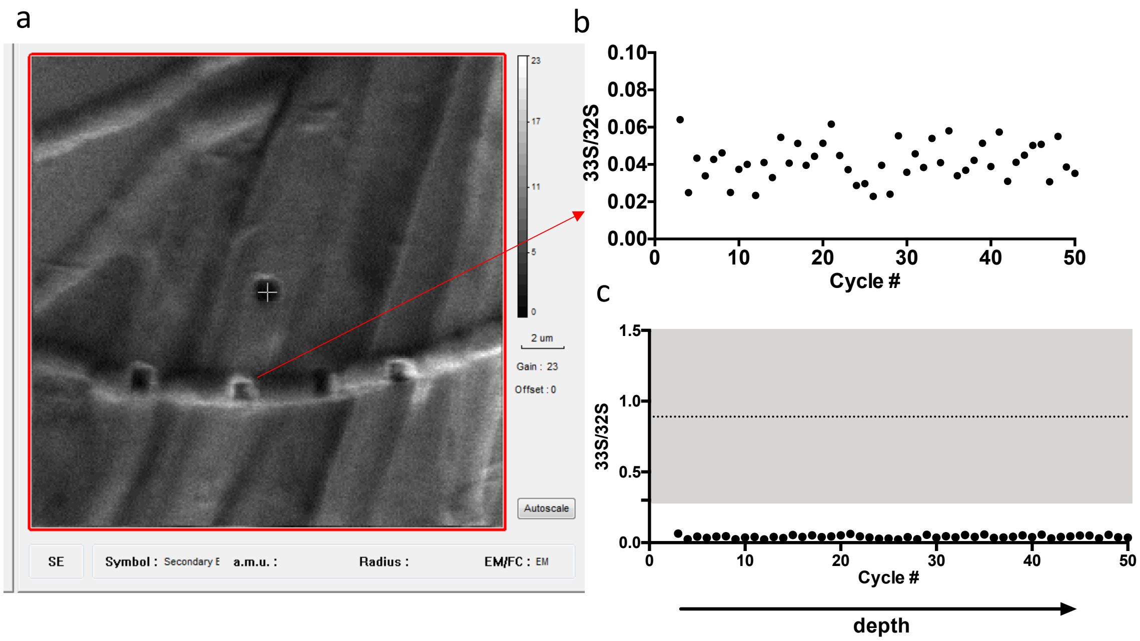

Supplementary Figure S2: NanoSIMS 1x1 μm^2 spot analysis. (a) Representative secondary electron image taken after multiple analyses of a *Nostoc* M2 filament on a moss phyllid. (b) Representative depth profile from indicated spot analysis, showing that the measured 33S/32S ratio does not systematically change with depth. (c) The same depth profile data points rescaled relative to the range of enrichment observed in the moss tissue at the corresponding time point, indicated by the shaded box. The dotted line shows the average ratio of moss tissue at this time point. The cyanobacterial ^{33}S enrichment is on average an order of magnitude lower than the moss tissue, and therefore, if the spot analysis sputtered through the cyanobacterial cell into the moss tissue, the depth profile data in panel (b) would increase significantly at depth. This was not seen in any of the cyanobacterial spot analysis depth profiles.

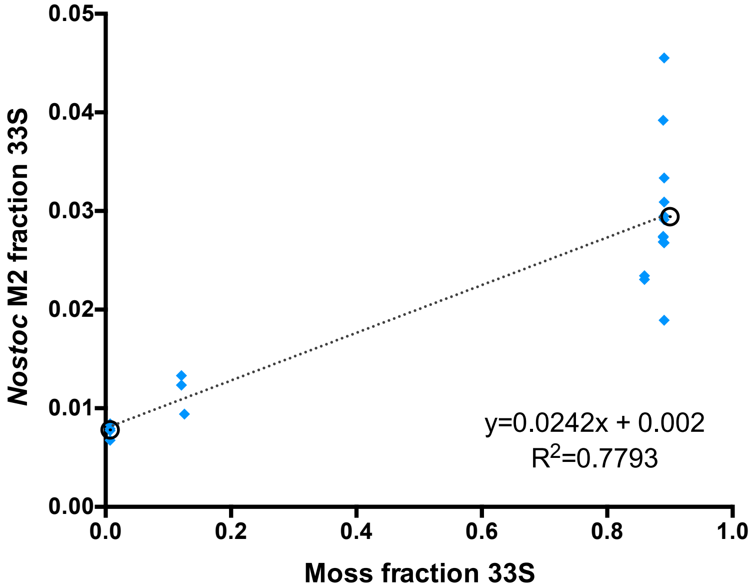

Supplementary Figure S3: NanoSIMS 1x1 µm^2 spot analysis of control samples to test for background ^{33}S counts from the surrounding tissue. Control samples are *Nostoc* M2 cells were grown with natural sulfur media, fixed with paraformaldehyde, and deposited on moss phyllids with on different levels of ^{33}S enrichment. The cyanobacterial cells were then analyzed for ^{33}S enrichment (here, f_{33S}) by spot analysis (blue points). The linear regression through the data (dotted line) was used to estimate the contribution of surrounding phyllid to the cyanobacterial spot analysis (~2.4%), which is the basis for our mixing model for the spot analyses: $0.024*f_{33S\text{-moss-meas}} + (1 - 0.024)*f_{33S\text{-cyano-est}} = f_{33S\text{-cyano-meas}}$. We used the measured ^{33}S fractions for the moss and cyanobacterial ($f_{33S\text{-moss-meas}}$ and $f_{33S\text{-cyano-meas}}$, respectively) and solved this mixing model for the estimate the true 33S fraction in each cyanobacterial cell ($f_{33S\text{-cyano-est}}$).

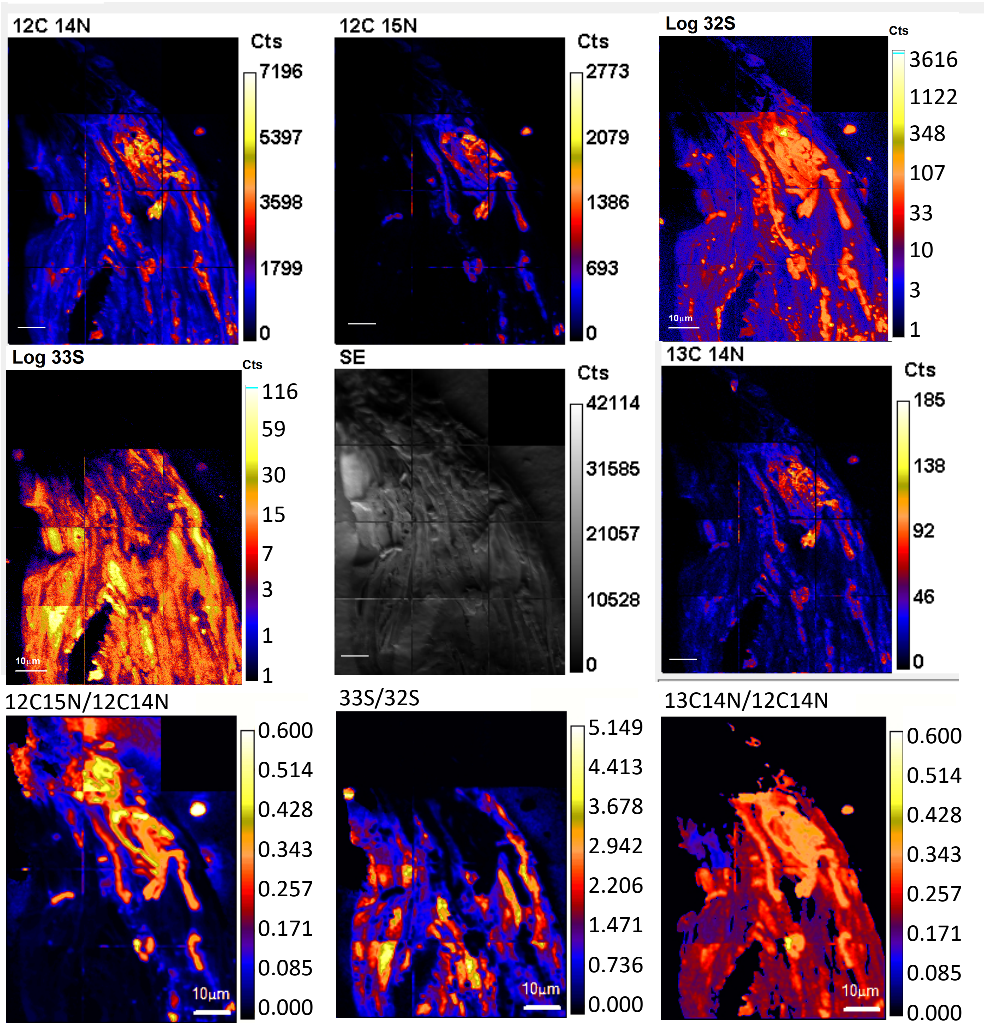

Supplementary Figure S4 NanoSIMS individual ion and ratio images for representative phyllid in Figure 3.

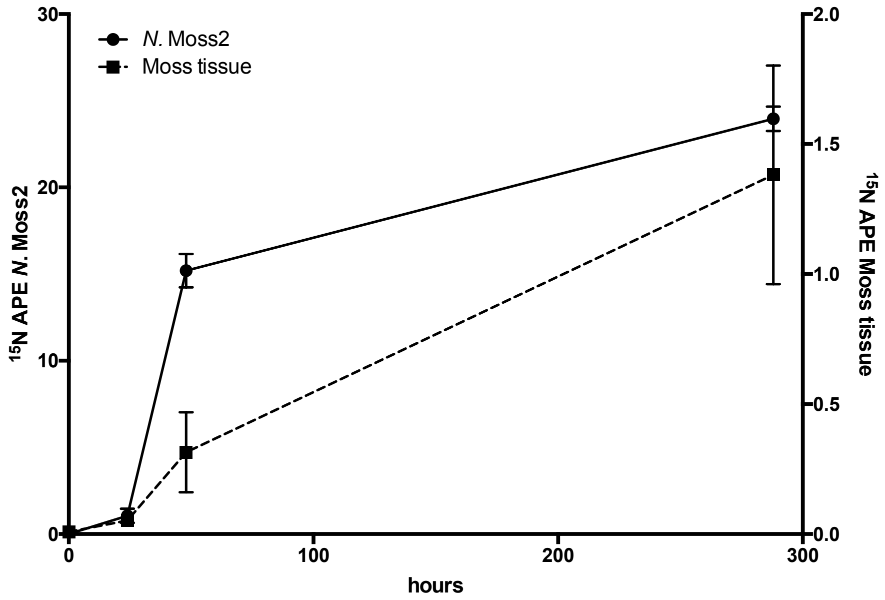

Supplementary Figure S5 NanoSIMS-derived N fixation during moss colonization and matching moss enrichment.

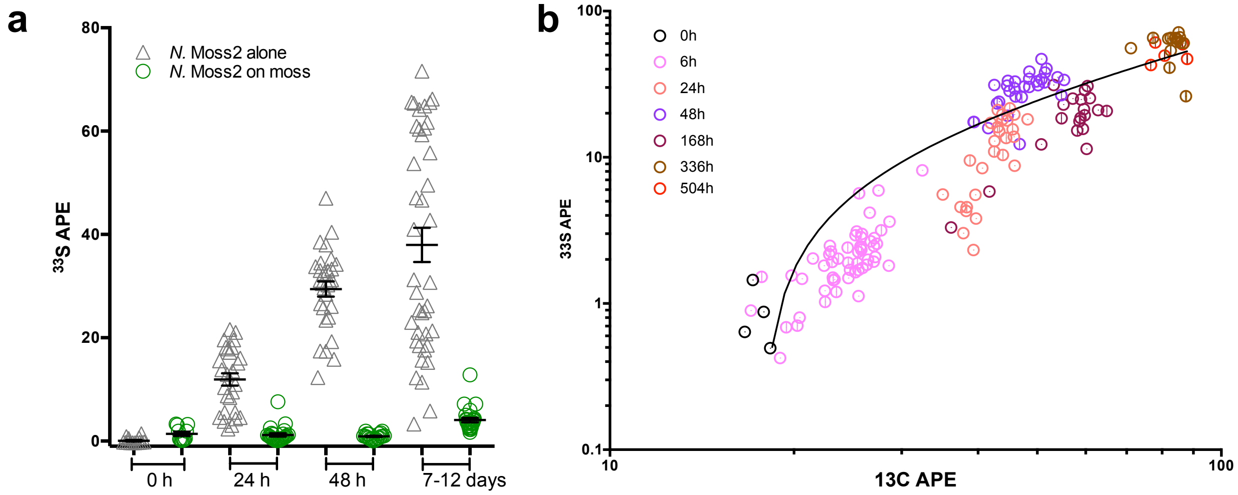

Supplementary Figure S6 NanoSIMS-derived *Nostoc* M2 single cell $Na_2{}^{33}SO_4$ and ^{13}C-bicarbonate incorporation in culture over time. (A) Each spot represents ^{33}S APE values of single *Nostoc* M2 cells from cultures (grey) or from moss colonization (green), over either 7 days (cultures) or 12 days (colonized). (B) Comparison of ^{33}S and ^{13}C incorporation over a 21-day incubation in single cells of *Nostoc* M2 in culture. Each spot represents ^{33}S APE and ^{13}C APE values of single *Nostoc* M2 cells at a given time point. Black line indicates a linear regression analysis across all points ($Y = 0.7566*X - 13.44$; $R^2=0.790$, $p<0.0001$).

Supplementary Table S1: Primers used in this study

Label	Sequence	Target
NpunKO-3.2	AGGATCTAGGTGAAGATCCTTTTTG	origin of transfer (oriT)
NpunKO-4	CGCGTGCTATAATTATACTAATTTTATAAGG	origin of transfer (oriT)
NpunKO-1	TCTAGAGGATCTCAATGAATATTGGTT	neomycin resistance gene cassette
NpunKO-2	CCCGGTGGGCGAAGAACTC	neomycin resistance gene cassette
NpunKO-21	TTTTTTCCTCCTTATAAAATTAGTATAATTATAG	
	CACGCGTGCGTAAAATTATCTTTGCA	*N. punctiforme* ΔF5882-KO1
NpunKO-22	ATACGCCCGTGTCAACCAATATTCATTGAGAT	
	CCTCTAGACATCTATATGTTTAGCAGCA	N. punctiforme ΔF5882-KO2
NpunKO-23	TGGAGTTCTTCGCCCACCGGGTTTATCTAAC	
	TTGGGGCGAACCTCCACAGCAAGTTGCTC	N. punctiforme ΔF5882-KO3
NpunKO-24.2	CATGAGATTATCAAAAAGGATCTTCACCTAGA	
	TCCTCAATAACTTTTCTGCTATACGGGA	N. punctiforme ΔF5882-KO4
rEP29	GGTCGGAGTCGCAGTCCGG	2059-R (sepF)
rEP30	CCCAGGCCATTCAGGCTTTG	2059-F (sepF)
gEP11	GTTAAGAAAAGCCCATAAAACGG	*N. punctiforme* ΔF5882-scrn1
gEP12	GTCTAGCTTGATCGCCGATG	N. punctiforme ΔF5882-scrn2

Supplementary Table S2: Headspace analyses

Sample description	12 CO2	13 CO2	13CO2 APE	H2O
24h	184.118 +/- 0.161	2.731 +/- 0.004	0.333 +/- 0.011	0.199 +/- 0.001
12 day	87.215 +/- 0.173	0.974 +/- 0.004	-0.023 +/- 0.009	0.187 +/- 0.001
no isotope 24h	143.636 +/- 0.178	1.615 +/- 0.004	-0.0155 +/- 0.01	0.196 +/- 0.001
Standard	669.948 +/- 0.281	7.469 +/- 0.004	-0.021 +/- 0.012	0.018 +/- 0.001
Standard	686.479 +/- 0.262	7.652 +/- 0.004	-0.022 +/- 0.012	0.015 +/- 0.002
Standard	688.855 +/- 0.277	7.678 +/- 0.004	-0.022 +/- 0.012	0.015 +/- 0.002
Standard	688.665 +/- 0.28	7.675 +/- 0.004	-0.022 +/- 0.012	0.012 +/- 0.001
Standard	689.027 +/- 0.259	7.68 +/- 0.004	-0.022 +/- 0.012	0.013 +/- 0.002
Standard	688.698 +/- 0.275	7.675 +/- 0.004	-0.022 +/- 0.012	0.010 +/- 0.001

Supplementary Table S3: Sulfur starvation genes

		Chemical		Physical	
Locus	Description	FC	p value	FC	p value
Npun_F5882	aliphatic sulfonate monooxygenase	-0.357	0.053	-0.853	0.0000
Npun_F5395	sulfate ABC transporter periplasmic sulfate-binding protein	-0.825	0.0000	-2.982	0.0000
Npun_R4761	sulfate ABC transporter periplasmic sulfate-binding protein	-0.422	0.0055	-0.782	0.0002
Npun_F5397	sulfate ABC transporter inner membrane subunit CysW	-0.708	0.0000	-0.51	0.0276
Npun_F5396	sulfate ABC transporter inner membrane subunit CysT	-0.662	0.0019	-1.041	0.0000
Ga0080672_1023025	sulfate transport system substrate-binding protein	-1.301	0.0000	-1.241	0.0000
Ga0080672_1023024	sulfate transport system permease protein	-0.896	0.0000	-0.447	0.0751
Ga0080672_1023023	sulfate transport system permease protein	-0.605	0.0002	0.343	0.1222
Ga0080672_102624	sulfate transport system ATP-binding protein	-0.938	0.0000	0.041	0.7887
Ga0080672_102995	sulfate transport system substrate-binding protein	-0.808	0.0009	-1.134	0.0213

Supplementary Methods

Growth conditions and isotope labeling prior to colonization

The cyanobacterial strains *Nostoc punctiform*e PCC 73102 , *Nostoc* M2 (Warshan *et al* 2017), *pks2*$^-$*, Nostoc* N996, and ΔF5882 (see below)**,** were grown in liquid $Bg11_0$ media (Rippka *et al* 1979). Colonization experiments and cultures were grown with constant shaking under 35 μmol photons m−2 s−1 at 19°C and 16h:8h light day cycle. Isotope labeling chambers were incubated at 21˚C, with 20 μmol $m^{-2}s^{-1}$ (4.16 W m^2) illumination, on a 12h:12h light:dark cycle. The slightly different photon flux and cycle from colonization experiments and isotope labeling incubations was due to different incubator equipment, and did not result in any visual effects on growth. For the stable isotope labeling and incubation experiments with ^{33}S, the BCD media had 1.01 M of both $Na^{33}SO^4$ (Sigma, 98+ atom%) and MgCl2*6H2O substituted for MgSO4*7H2O, which is normally present in the BCD media (Thelander *et al* 2007). *P. schreberi* was grown on ^{33}S substituted BCD agar media overlaid with filter paper for 42 days, through 3 rounds (approximately 2 weeks each) of blending, followed by another 2 weeks of growth, with filter transfer to new plates. For 18 days prior to experiment initiation, ^{33}S labeled *P. schreberi* (approximately 200 gametophytes) were incubated in a petri dish with agar BCD media in a sealed labeling chamber, 478 mL volume. CO_2 was scrubbed from the chamber with soda lime, and $^{13}CO_2$ (98+ atom %, Sigma) was injected at approximately 400 ppm (200 μl). 200 μl additional $^{13}CO_2$ was injected every other day for 18 days. Unlabeled control gametophytes were incubated in an identical manner excepting that natural abundance CO_2 was injected instead of $^{13}CO_2$.

Acetylene reduction assay

Nitrogen fixation rates were measured on triplicate 1 mL samples from 2 week old cultures placed in 10 mL chromatography vials equipped with rubber septa. 10% of the volume was replaced with acetylene and incubated for 4 hours, with above growth conditions, and ethylene production measured on Shimadzu GC-8A gas chromatograph (Shimadzu Corporation, Japan). Triplicate negative controls with $BG11_0$ media blanks were also run. Another set of triplicate 1 mL samples from the same cultures were pelleted and chlorophyll *a* extracted in cold absolute methanol and OD measured (Nagarkar and Williams 1997) using an Ultrospec 3000 spectrophotometer (Pharmacia Biotech, Cambridge, England).

Mutant construction

A plasmid to replace the putative alkanesulfonate monooxygenase (AMSO, Npun_F5882) was constructed as follows: Regions of the *N. punctiforme* chromosome 1-kb upstream and 1-kb downstream of approximately the midpoint of the targeted gene were amplified by PCR (Takara Bio, Mountain View, CA, USA) using primers specified in Supplementary Table S1. The neomycin resistance gene cassette was amplified using primers NpunKO-1 and NpunKO-2 and plasmid pRL448 as a template (Elhai and Wolk 1988). The 3' end of the 1-kb upstream PCR product and the 5' end of the 1-kb downstream PCR product had sequence homology to the neomycin resistance cassette to result in a final assembly product with the neomycin resistance flanked by the two regions of the gene to be interrupted. The vector, plasmid pRL2948a (C. P. Wolk, unpublished), provided an origin of transfer (oriT) for RP4-based conjugation, and a counter selectable sacB-erythromycin resistance gene (EmR). It was amplified using primers NpunKO-4 and NpunKO-3.2. Each product was purified by PCR cleanup kit (QIAquick, Qiagen). Each of the plasmids to knockout an *N. punctiforme* gene was assembled from four pieces (1-kb upstream, neomycin resistance gene, 1-kb downstream, and vector) using Gibson Assembly (Gibson *et al* 2009).

After production and segregation of the resulting line, *N. punctiforme* ΔF5882::Neo^{R} (ΔF5882), RT-PCR was performed using the KO-F5882-scrn1 and KO-F5882-scrn2 primers, confirming segregation (Fig. S1a). Additionally, RT-PCR of ΔF5882 RNA showed no evidence of gene expression from the Npun_F5882 locus (Fig. S1b), confirming successful knock out of this gene. RNA was extracted using the Plant RNeasy kit (Qiagen, Germany), and DNase treatment was performed using the on column RNase-Free DNase Set (Qiagen, Germany), both according to the manufacturer's instructions. RNA concentration was measured using the Qubit 2.0 Fluorometer (Life Technologies) with the RNA Assay Kit (Life Technologies). For the qRT- PCR cDNA synthesis was done using the RevertAidTM First Strand cDNA Synthesis Kit (Thermo-Scientific).

NanoSIMS analysis

All samples were stored in an argon dry box. Samples were mapped first with epifluorescence microscopy to identify *Nostoc* M2 cells, and then coated with ~5 nm of gold and imaged with a FEI Inspect F scanning electron microscope (Hillsboro, OR) to identify areas of interest for NanoSIMS imaging. A focused 2pA 150nm diameter 16 keV $^{133}Cs^{+}$ primary ion beam was scanned in a raster pattern (for images: 225-625 μm^{2} analyses areas, 256-by-256 pixels with a

dwell time of 1 ms/pixel for 10-30 cycles; for spot analyses: 1 μm^2 areas, 32-by-32 pixels with a dwell time of 3 ms/pixel for 10 cycles). To quantify $^{33}S/^{32}S$, $^{15}N/^{14}N$, and $^{13}C/^{12}C$ at each location, [$^{12}C^{14}N^-$, $^{12}C^{15}N^-$, $^{32}S^-$, $^{33}S^-$] and [$^{12}C^{14}N^-$, $^{13}C^{14}N^-$, $^{32}S^-$, $^{33}S^-$] were alternately collected using electrostatic peak jumping ("combined analysis"). Secondary electrons were also simultaneously collected. For imaging analysis, samples were first sputtered with 90 pA of Cs^+ current to an approximate depth of 50 nm to reach sputtering equilibrium (Ghosal *et al* 2008). Samples without any introduced stable isotope label (unlabeled control) were run to ensure no sources of extraneous label was introduced through samples preparation and analysis, to make sure samples of that material ran properly, and for calculations of atom percent excess (APE). The data are presented as atom percent excess (APE) (Pett-Ridge and Weber 2012, Popa *et al* 2007):

$$APE = \left[f_f - f_i\right] \cdot 100\% \,, \quad (1)$$

where f_f and f_i are the final and initial fractions of the spiked isotope in the sample. For N and C, which are two isotope systems, these abundances are calculated using $f = R/(R+1)$, where R is the measured ratio. To accurately calculate ^{33}S APE from the measured ratio $^{33}S/^{32}S$, the abundance of ^{34}S must be included:

$$f_{33S} = \frac{\left(^{33}S/^{32}S\right)_{measured}}{\left(^{33}S/^{32}S\right)_{measured} + 1 + \left(f_{^{34}S}/f_{^{32}S}\right)_{natural}} \,, \quad (2)$$

where $f_{^{34}S}$ = 0.042 and $f_{^{32}S}$ = 0.95. This formulation is based on the relative abundance of ^{34}S to ^{32}S being fixed, ^{34}S being negligible in the $Na^{33}SO_4$, and ^{36}S being negligible in all cases.

References

Elhai J, Wolk CP (1988). Conjugal transfer of DNA to Cyanobacteria. *Methods Enzymol* **167:** 747-754.

Ghosal S, Fallon SJ, Leighton TJ, Wheeler KE, Kristo MJ, Hutcheon ID *et al* (2008). Imaging and 3D Elemental Characterization of Intact Bacterial Spores by High-Resolution Secondary Ion Mass Spectrometry. *Anal Chem* **80:** 5986-5992.

Gibson DG, Young L, Chuang R-Y, Venter JC, Hutchison III CA, Smith HO (2009). Enzymatic assembly of DNA molecules up to several hundred kilobases. *Nat Methods* **6:** 343.

Nagarkar S, Williams GA (1997). Comparative techniques to quantify cyanobacteria dominated epilithic biofilms on tropical rocky shores. *Mar Ecol-Prog Ser* **154:** 281-291.

Pett-Ridge J, Weber PK (2012). NanoSIP: NanoSIMS applications for microbial biology. *Microbial Systems Biology*. Humana Press. pp 375-408.

Popa R, Weber PK, Pett-Ridge J, Finzi JA, Fallon SJ, Hutcheon ID *et al* (2007). Carbon and nitrogen fixation and metabolite exchange in and between individual cells of *Anabaena oscillarioides*. *ISME J* **1:** 354-360.

Rippka R, Deruelles J, Waterbury JB, Herdman M, Stanier RY (1979). Generic Assignments, Strain Histories and Properties of Pure Cultures of Cyanobacteria. *J Gen Microbiol* **111:** 1-61.

Thelander M, Nilsson A, Olsson T, Johansson M, Girod P-A, Schaefer DG *et al* (2007). The moss genes PpSKI1 and PpSKI2 encode nuclear SnRK1 interacting proteins with homologues in vascular plants. *Plant Mol Biol* **64:** 559-573.

Warshan D, Espinoza JL, Stuart RK, Richter RA, Kim S-Y, Shapiro N *et al* (2017). Feathermoss and epiphytic *Nostoc* cooperate differently: expanding the spectrum of plant-cyanobacteria symbiosis. *ISME J.*

This work was performed under the auspices of the U.S. Department of Energy by Lawrence Livermore National Laboratory (under contract DE-AC52-07NA27344).
